# Clinical Outcomes of Three versus Four Mini-Implants Retaining Mandibular Overdenture: A 5-Year Randomized Clinical Trial

**DOI:** 10.3390/medicina60010017

**Published:** 2023-12-21

**Authors:** Asja Celebic, Ines Kovacic, Nikola Petricevic, Mohammed Nasser Alhajj, Jolanda Topic, Luka Junakovic, Sanja Persic-Kirsic

**Affiliations:** 1 Department of Removable Prosthodontics, School of Dental Medicine, University of Zagreb, 10000 Zagreb, Croatia; petricevic@sfzg.hr (N.P.); persic@sfzg.hr (S.P.-K.); 2 Department of Prosthodontics, Faculty of Dentistry, Thamar University, Thamar 87246, Yemen; m.n.alhajj@hotmail.com; 3 Private Dental Office, 21300 Makarska, Croatia; dr.jolanda.topic1@gmail.com; 4 School of Dental Medicine, University of Zagreb, 10000 Zagreb, Croatia; 5 Mag. Math., Libertas International University, 10000 Zagreb, Croatia; luka.junakovic@gmail.com

**Keywords:** mini-implants, mandibular overdenture, marginal bone level, success, survival

## Abstract

*Background and Objectives*: Due to a lack of long-term clinical studies that would clarify whether the insertion of three mini-implants (MDIs) can be as successful treatment as the insertion of four MDIs for the retention of mandibular overdentures (ODs), this 5-year prospective cohort study was set up. *Materials and Methods:* Participants (*n* = 83) randomly received either four or three MDIs and a mandibular OD. Clinical examinations were performed at the baseline, as well as after one, three, and five years, respectively. A total of 73 participants (38 in the four-MDI and 35 in the three-MDI groups) partook in the study. The marginal bone level change, success and survival rates, and prosthodontic maintenance were assessed. *Results:* Repeated measures showed that the mean peri-implant bone loss increased progressively at a small amount over five years in both groups (four-MDI group = −0.36 ± 0.74; three-MDI group = −0.33 ± 0.27 mm; *p* < 0.05). However, an ANCOVA revealed no significant effects of the group (no significant difference between the three- and the four-MDI groups; F = 0.085; *p* = 0.771), gender (F = 0.023; *p* = 0.88), or covariate age (F = 1.95; *p* = 0.167) on the dependent variable: the 5-year MBL change. The success rate (together with successful survival) was 93.8% in the four-MDI group and 91.7% in the three-MDI group. The log-rank (Mantel–Cox) test revealed no significant differences between them (*X*^2^ = 0.373; *p* = 0.541). *Conclusions:* In patients with narrow ridges, the insertion of three MDIs in the mandible for overdenture retention can be equally as successful as the insertion of four MDIs.

## 1. Introduction

Mini dental implants (MDIs) with an active surface for osseointegration have been on the dental market for over 20 years as an alternative to standard-sized implants, because their reduced diameter enables their insertion into alveolar ridges with a reduced buccolingual width without the need for bone augmentation [1,2,3,4,5]. They are mostly made from the Ti-6Al-4V titanium alloy and have grade 5 mechanical properties. Although MDIs were once considered to be implants with a diameter less than 3.0 mm [6], the newest classification defines them as one-piece category 1 narrow implants with a diameter ranging from 1.8 to 2.5 mm [7]. The insertion of MDIs can often be performed without raising a flap, thus decreasing the duration of postsurgical recovery [8,9]. The insertion of four MDIs in the mandible for complete overdenture (OD) retention has been verified as an excellent clinical treatment option in subjects with narrow alveolar ridges, due to the very good survival and success rates of implants accompanied by increased patient satisfaction [3,4,5,10,11,12,13,14]. Even the insertion of four short MDIs (length ≤ 8 mm) and a mandibular OD was a successful treatment option in extreme mandibular atrophy [15,16]. The fixed-type MDIs have proved to be successful in the retention of crowns or short bridges in the mandibular incisor region [17]. Furthermore, the insertion of two or more MDIs for the retention of partial removable dentures was also confirmed as a successful treatment modality [18,19,20].

The question raised in this study was whether the retention of a mandibular OD with three MDIs can be as successful as with four MDIs. No prospective clinical studies have been conducted to test whether three MDIs can sufficiently retain and support mandibular ODs over a longer period. Therefore, the aim of this randomized, controlled prospective clinical trial was to assess the success of using only three MDIs for mandibular OD retention and comparing them with four MDIs. This study measured peri-implant marginal bone level (MBL) change and success and survival rates, comparing the three-MDI group with the control subjects, which were in fact, the cohort of patients with four MDIs (four-MDI group) over the period of five years. 

Marginal bone loss refers to the gradual reduction in bone height around the implant site. While some degree of bone remodeling is natural, excessive marginal bone loss can impact the long-term success of dental implants. Various factors contribute to this phenomenon including surgical techniques, implant design, infection, loading protocol, extent of chewing forces, and other individual patient characteristics [21,22,23,24] The null hypothesis was that no significant differences in MBL changes and success and survival rates would be found between the three- and four-MDI groups.

## 2. Materials and Methods

### 2.1. Study Design

This prospective randomized clinical cohort study was performed between September 2015 and October 2022 at the School of Dental Medicine, University of Zagreb, Croatia, with the approval of the institution’s ethical committee (No. 05-PA-26-6/2015). The study protocol was in compliance with the Declaration of Helsinki. Before obtaining signed informed consent documentation, all procedures and possible complications, risks, and benefits were explained in detail to each participant. 

### 2.2. Inclusion and Exclusion Criteria

An edentulous mandibular alveolar ridge width < 5.5 mm in the interforaminal region was the determining factor for inclusion in this study. Participants with wider ridges were excluded, as they could receive standard-sized implants. The available bone volume and length were measured on preoperative CBCT scans and digital panoramic radiographs. All participants were also required to have a eugnathic jaw relationship in their centric position. General exclusion criteria were not different from those accepted for any implant placement and were based on general health issues [25]. Local exclusion criteria were as follows: attached mucosa of a denture-bearing area thicker than 4.0 mm, flabby ridge, mandibular bone height less than 16 mm in the interforaminal region, participants having dysgnathia, and a history of radiotherapy in the mandible or neighboring regions. 

Randomization in assigning edentulous participants into the 3- or 4-MDI groups was completed by assigning odd and even numbers to the participants. The first participant was assigned an odd number, the second an even number, and so on. Each participant assigned an odd number received 4 MDIs, while each participant assigned an even number received 3 MDIs in the mandible. 

However, the surgeons who inserted the implants were not blinded to the procedure; neither were the prosthodontists who manufactured and loaded the overdentures. The costs of MDIs were covered by the research grant No. 1218/2014 (Croatian Science Foundation), and the costs of mandibular ODs were covered by the health insurance of each participant. All MDIs were inserted and all new ODs were manufactured during the first two years of the study (from October 2014 to December 2016).

### 2.3. Sample Size Calculation

The sample size calculation was made based on the primary outcome, i.e., peri-implant MBL change. Assuming that the mean MBL change may differ up to one mm between the groups with a standard deviation of one mm in each group and accounting for even 30% of possible dropouts during the observation period (five years), the calculation showed that 26 participants should be included in each group, with the type I probability error alpha set at *p* < 0.05 and the power at 80%. However, at the baseline of the study, a total of 83 participants were included: 42 in the 4-MDI group and 41 in the 3-MDI group.

### 2.4. Surgical Procedures

The inserted MDIs were ball-type (Dentium, South Korea) and 2.0 or 2.5 mm wide and 10–14 mm long, depending on the available bone volume assessed on the preoperative CBCT scans. The two experienced specialists in oral surgery, both with >10 years of experience, inserted the MDIs. 

**Figure 1:**
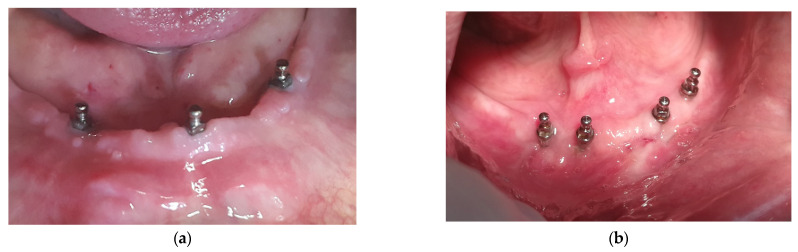
Participants with three (**a**) or four mini dental implants (MDIs) (**b**) inserted in the mandible for retention of mandibular overdentures.

Participants in the 3-MDI group received 3 ball-type MDIs. Two posterior MDIs were inserted in the approximate region previously occupied by the distal surfaces of the canines. The third MDI was inserted at the midline, or as close as possible to the midline of the mandible (Figure 1a). Participants in the 4-MDI group received 4 ball-type MDIs in the sites of their previous first premolars (two posterior MDIs) and second incisors (two anterior MDIs) (Figure 1b). 

Surgical procedures were performed by two experienced surgeons after consulting a specialist in prosthodontics. The surgical techniques performed were the flapless or the open flap techniques, depending on the morphology and volume of the available bone. The open-flap technique was utilized when a pointed narrow ridge had to be leveled, or the mucosa of a denture-bearing area needed alterations. In cases when the flapless method was used, the first step was to punch a small amount of oral mucosa into the preparation site to ensure a stable drill position. A physiodispenser (W&H Implantmed, GmbH, Salzburg, Austria) and an external drill cooled by a saline solution were used. The bone was prepared using the pilot and final drill (Dentium drilling set); however, the final drill diameter was always smaller than the MDI diameter (1.3–1.8 mm for 2.0 mm wide MDIs; 1.8–2.3 mm for 2.5 mm wide MDIs). The depth of preparation was determined depending on the bone quality. The preparations were made one, two, or three mm shorter than the implant length (longer preparations were performed in a denser bone). The only exception was when the MDI had to end in the dense lower mandibular cortex; in that case, the whole mini-implant length had to be prepared, as the tip of the MDI could not advance into the dense D1 bone. Antibiotics were administered one hour before surgery for prophylactic reasons (2 g amoxicillin with clavulanic acid or 600 mg clindamycin). Local anesthesia was administered prior to the surgery (Ubistesine forte 4% or Mepivastesin 3%, 3M). Each MDI was inserted into the preparation site and rotated clockwise, exerting a downward pressure (self-tapping insertion technique). The implants were transferred into the preparation hole with a plastic carrier from the original package and rotated until the plastic carrier broke; then, the thumb wrench was used and finally, the torque wrench. The whole roughened threaded MDI surface had to be inserted into the bone. The transmucosal part of the smooth MDI profile emerged from the attached mucosa into the oral cavity with the platform and the ball-type head for OD retention. The final MDI insertion torque values varied between 30 and 55 Ncm in both groups (3-MDI and 4-MDI groups, respectively).

All patients were given the following postsurgical instructions: no alcohol or smoking for two days, no hot beverages, ice packs for cooling, antiseptic mouth rinse (chlorhexidine gluconate 0.12%), and analgesics when necessary (nonsteroid anti-inflammatory drugs). Detailed instructions on how to maintain oral hygiene were also provided.

### 2.5. Prosthodontic Protocol 

All new mandibular ODs were made by two trained specialists in prosthodontics and were delivered two to three months after the MDI insertions. The procedure was as follows: After obtaining alginate impressions, custom trays were made to obtain custom impressions for each participant. They were made using a thermoplastic border material and an additive low-viscosity silicone. The occlusal rims were made in the laboratory. The vertical jaw dimension of the lower jaw was recorded at the centric jaw relation for each participant and transferred into a semi-adjustable articulator. Semi-anatomical artificial teeth (lingualized occlusion scheme) were set up in the laboratory. After testing a trial denture to verify satisfactory esthetics and antagonistic contacts in the centric relation, new dentures were processed. All mandibular ODs were strengthened with a CoCr metal framework to prevent fractures. One to two days after the OD delivery and trial wearing (without MDI loading), metal housings with O-rings were mounted chairside using blockout shims and a self-curing acrylic resin (GC Reline, GC America Inc., Alsip, IL, USA). The occlusion was again checked and adjusted. During the adaptation period (fifteen days), the oral mucosa was inspected for soreness, and the denture was trimmed off if necessary. 

### 2.6. Radiographic Evaluation 

**Figure 2:**
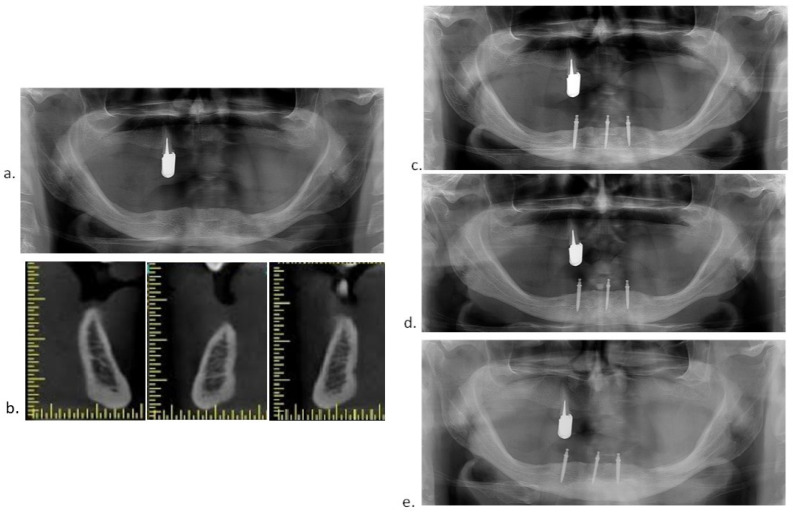
(**a**) Preoperative panoramic radiograph; (**b**) preoperative CBCT; (**c**) panoramic radiograph after insertion of 3 MDIs; (**d**) panoramic radiograph obtained at the 3-year follow-up examination; (**e**) panoramic radiograph obtained at the 5-year follow-up examination.

Preoperative CBCT scans and digital panoramic radiographs were obtained to determine mini-implant dimensions. Panoramic radiographs were obtained again after the MDI insertions to check their parallelism and positions. In some participants, panoramic radiographs were also obtained at the follow-up examinations (Figure 2a–e).

**Figure 3:**
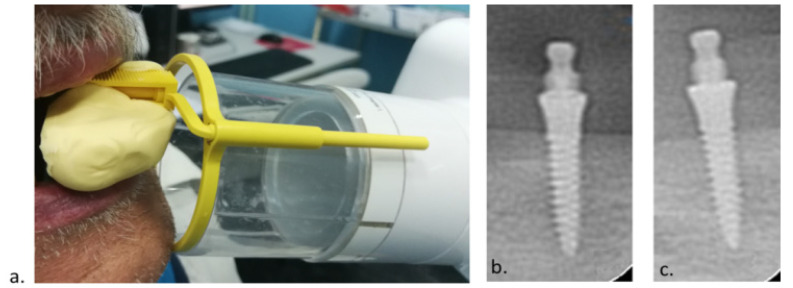
(**a**) Silicone index and the long-cone paralleling technique; (**b**) postoperative radiogram; (**c**) 5-year follow-up radiogram.

At the MDI loading (mounting of metal housings with O-rings), digital retroalveolar radiographs were obtained to determine the marginal bone level (Minray Soredex Intraoral, Tuusula, Finland, 70 kV, 0.16 mAs; the long-cone paralleling technique). The digital retroalveolar radiographs were also obtained at the 1-, 3-, and 5-year clinical follow-up examinations. A film holder (X-ray holder, Super-Bite^®^, Kerr USA, Orange, CA, USA) with a customized silicone index for each patient was used for reproducibility (Figure 3a–c).

The MBL change was measured at the mesial and distal sites of each MDI using Scanora^TM^ software 5.1. (Soredex, Tuusula, Finland) at a 10× magnification. The values were rounded to the nearest 0.1 mm. In cases where a part of the smooth MDI surface was submerged into the bone during insertion, bone loss, until it reached the roughened threaded surface, was considered as bone remodeling, not as bone loss. Radiographic evaluation was performed by two experienced specialists in prosthodontics who were not aware of the group classification.

### 2.7. Implant Success and Survival Rates

Implant success, survival, or failure rates were also assessed at the 1-, 3-, and 5-year follow-up examinations. The assessments were based on the Consensus Conference of the International Congress of Oral Implantology held in Pisa in 2007 [26]. Implants were categorized into successful, survival, or failure groups. Implant survival was categorized either as satisfactory or compromised survival. Successful implants were those where participants had no ongoing pain (or a history of pain), no dysesthesia or a foreign body sensation, no peri-implant infection, and no mobility when the peri-implant radiolucency was less than 2 mm and when the implant was suitable for a prosthodontic restoration. Satisfactory survival was described as a peri-implant marginal bone loss slightly > 2 mm either at the mesial or distal site, but not requiring any clinical management. Any implants requiring serious clinical treatment to reduce the risk of failure were listed in the compromised survival category. The MDIs were listed in the failure category when they required removal or had been lost already.

### 2.8. Prosthetic Complications and Maintenance

Mandibular OD base fractures, a need for relining, loosening of metal housings, or artificial teeth detachment were considered as complications, while the replacement of retentive elements (“O”-rings) was considered as maintenance.

### 2.9. Statistical Analysis

Statistical analysis was performed by using SPSS 20.0 software. A one-sample Kolmogorov–Smirnov test was used to test the normality of the MBL change distribution. An *X*^2^ test was used to test the significance of gender differences between the 3- and 4-MDI groups. An independent sample *t*-test was used to test the significance of the differences between the 3- and 4-MDI groups at each time point of observation (1, 3, and 5 years). The repeated-measures analysis was performed for the MBL changes between the three time points (1, 3, and 5 years, respectively) in both groups (the 3- and the 4-MDI groups). An ANCOVA was performed to test the significance of the differences for the variable MBL change, with fixed factors: the group (3 or 4 MDIs), gender (female or male), and age as covariates. 

Kaplan–Meier curves were utilized for the survival analysis (MDI failures and compromised survivals were counted as one category, while successful implants and satisfactory survivals were counted together as another category). A comparison of the 4-MDI and 3-MDI groups was made using the Log-rank Mantel–Cox test.

## 3. Results

**Figure 4:**
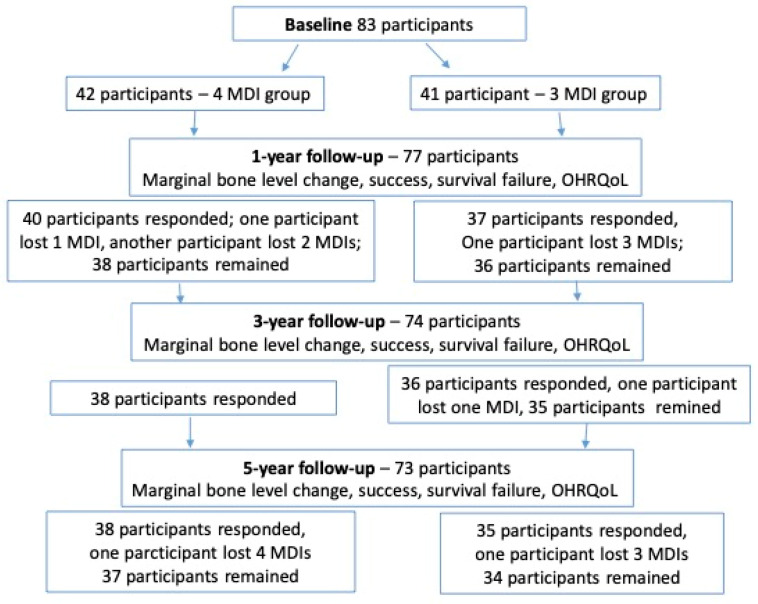
Flow diagram of the study.

From the baseline of 83 participants in both groups, a total of 73 subjects completed the 5-year observation. The flow diagram of the participants is shown in Figure 4. The participants who responded to the 5-year recall clinical examination consisted of 38 subjects in the four-MDI group and 35 subjects in the three-MDI group. The four-MDI group comprised 24 females, and the three-MDI group comprised 17 females; no gender difference was found (*X*^2^ = 1.57, df = 1, *p* = 0.21). The groups were also not different according to the age of the participants (t = 0.52, df = 75; *p* = 0.61). The mean age ± standard deviation in the four-MDI group was 68.3 ± 9.97 years, while the mean age in the three-MDI group was 69.35 ± 7.71 years. Two participants were lost to follow-up in the four-MDI group after one year, and four participants were in the three-MDI group, so seventy-seven participants remained after one year. Furthermore, in the four-MDI group, one participant lost one MDI, and another participant lost two MDIs during the first year (two MDIs were 2.0 mm wide, one was 2.5 mm wide), while in the three-MDI group, one participant lost all three MDIs (two MDIs were 2.0 mm wide, one was 2.5 mm wide). At the 3-year examination, no more implants were lost in the four-MDI group (38 participants), while one implant fractured in the three-MDI group (2.0 mm wide) (35 participants remained). After 5 years, one participant lost all four implants in the four-MDI group (two were 2.0 mm wide; two were 2.5 mm wide) (they were so movable that they required removal and were categorized as failures), and one participant lost all three mini-implants in the three-MDI group (two of them fractured (2.0 mm wide) and the third one was movable (2.5 mm wide)). Finally, after the 5 years of mandibular OD-wearing, a total of 37 participants remained in the four-MDI group, and 34 remained in the three-MDI group.

### 3.1. Marginal Bone Level (MBL) Change

There was no significant difference in the mean marginal bone loss between the two posterior and two anterior implants in the four-MDI group at the 5-year follow-up examination (t = 1.88; *p* = 0.07). Also, there was no significant difference in the mean marginal bone loss between the two posterior and midline implants (t = 1.34; *p* = 0.19) in the three-MDI group. Mean values of the MBL changes after the first, third, and fifth year of OD wearing, together with the significance of the differences between the three- and the four-MDI groups are presented in Table 1. Levene’s test for equality of variances showed equal variances, and the t-test for independent samples showed no significant difference in the MBL change between the three- and the four-MDI groups at any of the observation stages (*p* > 0.05). 

In the three-MDI group (Wilks’ lambda = 0.517; F(2.32) = 15.4; *p* < 0.001; η^2^ = 0.233) as well as in the four-MDI group (Wilks’ lambda = 0.807; F(2.35) = 4.3; *p* = 0.021; η^2^ = 0.483), the peri-implant MBL change decreased progressively (for a small amount) over the observation period.

The ANCOVA (dependent variable = marginal bone change at the 5-year follow-up) revealed that the model was not significant (F = 0.68, *p* = 0.61), the group (three- or four-MDI) had no significant effects (F = 0.085; *p* = 0.771), nor did the gender (F = 0.023, *p* = 0.88) or the covariate age (F = 1.95; *p* = 0.167) on the rate of the 5-year marginal bone loss. The mutual effect of belonging to either the three- or the four-MDI group, or a different gender group was also not significant (F = 0.077; *p* = 0.78). Although not statistically significant (*p* > 0.05), at the 5-year observation stage, the mean MBL decrease was slightly negatively correlated with the age in the four-MDI (r = −0.15, *p* = 0.34) as well as in the three-MDI groups (r = −0.24, *p* = 0.19).

### 3.2. Success, Survival, and Failure Rates 

**Figure 5:**
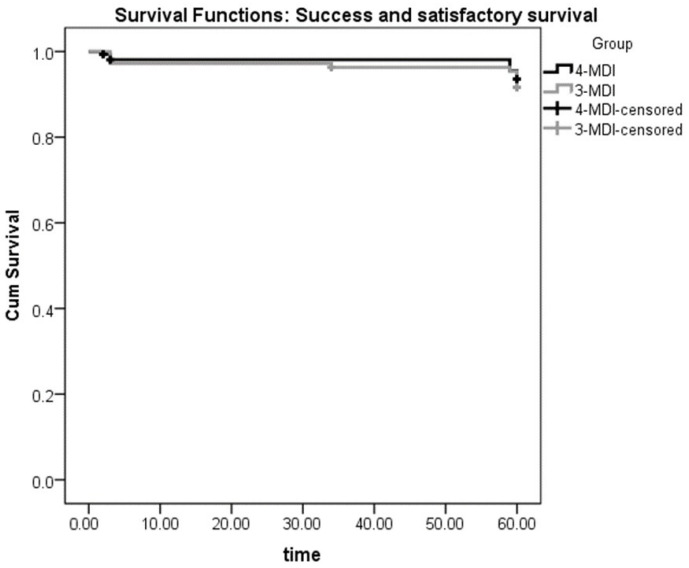
Implant-level Kaplan–Meier curves for success and satisfactory survival of 4 or 3 mini dental implants (MDIs) supporting mandibular overdentures over 5 years of follow-ups.

**Figure 6:**
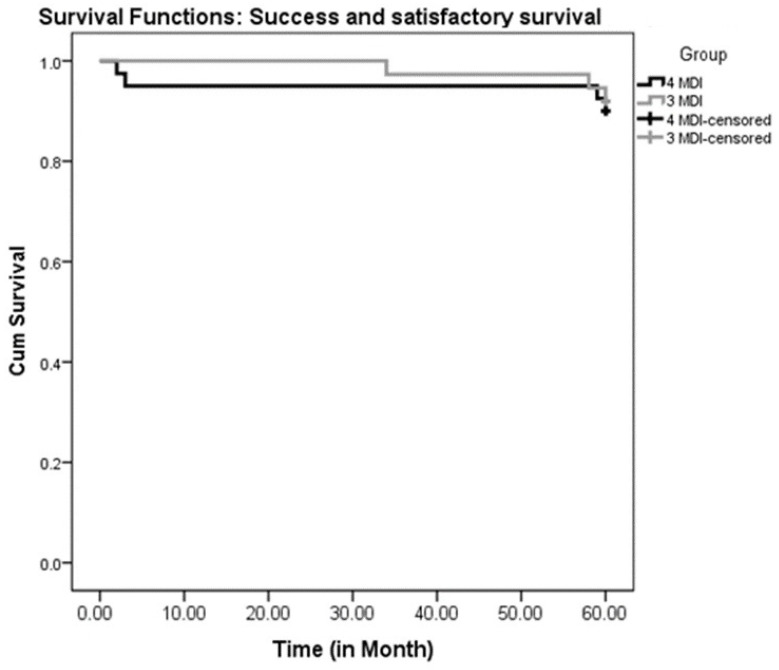
Patient-level Kaplan–Meier curves for success and satisfactory survival of 4 or 3 mini dental implants (MDIs) supporting mandibular overdentures over 5 years of follow-ups.

Success and satisfactory survival rates were analyzed together as one category, while failure and compromised survival were analyzed together as another category. The survival analysis comparing the four-MDI and the three-MDI groups throughout five years at the implant level is presented in Table 2 and Table 3. The survival analysis comparing the four-MDI and the three-MDI groups at the patient level throughout the same period of observation is presented in Table 4 and Table 5. The log-rank (Mantel–Cox) showed no significant difference between the three- and the four-MDI groups (*p* > 0.05). The Kaplan–Meier curves are presented in Figure 5 (MDI level) and Figure 6 (patient level). 

### 3.3. Prosthetic Complications and Maintenance

No OD fracture was registered either in the three- or the four-MDI group. The complications and the need for maintenance registered with mandibular ODs are presented in Table 6. At the 1-year observation stage, two ODs required relining: one in the three- and another in the four-MDI group, respectively. The same occurred after three years, while at the 5-year examination stage, three ODs required relining in the four-MDI group, and four ODs required relining in the three-MDI group. Seven “O”-rings were lost or needed to be changed in the three-MDI group and another three “O”-rings in the four-MDI group during the first year. During the second and third years, an additional 34 “O”-rings had to be changed in the three-MDI group and 24 in the four-MDI group. Up to the fifth year of observation, an additional 30 “O”-rings had to be changed in the three-MDI group and 24 in the four-MDI group, respectively. At the 5-year observation stage, all “O”-rings were changed in both groups. 

## 4. Discussion

Due to a lack of clinical evidence on whether the insertion of only three MDIs can be as equally successful of a treatment as the insertion of four MDIs for the retention of mandibular ODs over a longer period of time, this cohort study was set up. The 5-year results revealed small rates of peri-implant bone loss in both the three- and four-MDI groups, with no significant difference between them. The 5-year success and survival rates were also not different between the groups and were acceptable in both groups. Thus, the hypothesis was accepted.

The short time of clinical evidence (one year) showed that either two or four MDIs led to better oral health-related quality of life (OHRQoL), compared with two standard implants [5]. However, the costs were the least for the two mini-implant OD options [27]. One study (limited sample size) pointed towards increased patient satisfaction during the 7-year clinical observation with two MDIs [28]. However, the rate of MBL change was not presented. Another study (11 patients) showed a 1-year satisfactory masticatory efficiency with two MDIs [29]. On the other hand, Jofre et al. [30] showed a high amount of marginal bone loss in two unsplinted MDIs retaining a mandibular OD during the 15 months of clinical follow-up (1.40 +/− 1.02 mm), compared with the two MDIs splinted with a prefabricated bar (0.84 +/− 0.66 mm). Therefore, they recommended the splitting of two mini-implants for better clinical success or the insertion of four MDIs as single units. Also, a lack of data exists about the aftercare costs and survival rates of two mini-implants for mandibular OD retention [31]. The study of Misfud et al. [32] described an MBL change in two MDIs, but they used category 2 narrow implants (diameter = 2.9 mm, with locator system for the retention of ODs). As opposed to the insertion of only two MDIs as the less expensive treatment modality, Mangano et al. [33] reported that the insertion of either three or four MDIs in 62 patients was satisfactory in the 4-year prospective study (overall cumulative survival rate was 96.9%, and there were only 6% biologic complications). However, Magnano et al. [33] did not compare the outcomes of the three and the four MDIs. 

Our previous clinical experience showed that patients with four MDIs, even when they lost one implant, continued to wear dentures retained on only three MDIs. Therefore, as the less expensive treatment than the four MDIs, we chose to follow-up prospectively the option of inserting only three MDIs. The insertion of four MDIs is the standard of care for mandibular OD retention in edentulous subjects with narrow ridges [2,3,4,5,6,10,11,12,13,14,15,16,27,33,34,35,36,37,38,39,40,41,42]. The present 5-year cohort study compared clinical outcomes of the insertion of three MDIs with the outcomes of four MDIs for mandibular OD retention. No statistically significant differences were found between the groups over the intermediate-term clinical observation period of five years in mean peri-implant MBL change (−0.36 mm in the three-MDI group vs. −0.33 mm in the four-MDI group) and the success and satisfactory survival rates (93.8% in the four-MDI, and 91.7% in the three-MDI group, respectively). One study based on the review of the literature (1–7 years) reported that the mean survival rate of patients with four MDIs was 92.32% [34], while another similar study reported that the mean survival rate of four MDIs (category 1 narrow implants) was 94.7 ± 5% [35]. One study reported survival rates of 95.63% over the mean period of 28 months [10]. The overall survival rate (for four MDIs) after 5 years of treatment was 90.7% in the study of Hussein and Alruthea [36]. Enkling et al. [11] reported the 100% survival rate of four MDIs and ascribed it to the open-flap surgical approach at the baseline. The present study revealed similar (sometimes even better) survival rates to those listed in the literature [10,34,35,36] both for the 3- and the 4-MDI option. The study of Mundt et al. [37] reported 0.5 mm of peri-implant bone loss over the mean observation time of 2.2 ± 1.0 years with mandibular ODs loading four MDIs. Zygogiannis et al. [34] reported a one-year mean MBL change of −0.42 ± 0.56 mm in the mandibles with four MDIs. Another study [36] reported a mean peri-implant vertical bone loss of 1.61 ± 0.371 after 5 years. Our 5-year study revealed less bone loss in both groups (−0.33 ± 0.27 mm in the three-MDI group and −0.36 ± 0.74 in the four-MDI group). The lower amount of peri-implant vertical bone loss recorded in the present study can be attributed to the fact that bone loss in the submerged MDIs around the polished surface was considered as bone remodeling [15,17,19]. Only a decrease in the bone below the roughened, threaded surface was considered as bone loss. In this study, the MDIs were inserted during the open-flap surgery whenever it was necessary (the morphology of the available bone required bone leveling), which is in compliance with the study of Enkling et al., who reported a 100% success rate over five years when MDIs were inserted through open-flap surgery [11,39]. 

It is known that younger patients develop higher occlusal forces and chew more vigorously, while implants have less proprioception than periodontal ligaments [39]. That may account for a slightly negative (although not significant) correlation between the age and MBL decrease after five years, as found in this study [40]. When we consider maintenance in the three- and four-MDI groups, no fractures of denture bases were registered during the 5-year observation due to reinforcement by a metal Co–Cr framework in all delivered dentures. Most of the “O”-ring replacements had to be completed due to calculus formation (lost elasticity) or wear; only a few “O”-rings fell out of metal housings in both groups. Although the manufacturer recommends changing the “O”-rings once a year, only a few “O”-rings had to be changed after one year, similar to other studies [38,39,41]. A slightly larger number of “O”-rings had to be replaced in the three-MDI group during the 5-year period. All “O”-rings were only changed after five years, which is consistent with another study on MDIs utilized for partial removable denture retention [19].

The keratinized mucosa thickness can influence the amount of marginal bone loss. It was reported that the peri-implant bone loss is lesser in thick than in thin mucosa biotypes [42,43,44]. However, we did not measure the height of the peri-implant mucosa, which is one of the study limitations. 

The limitations of the present study are the 2-dimensional assessment of peri-implant bone level, variations in the amount of chewing force between the participants, variations in the diameter (2.0–2.5) and length (10, 12 or 14 mm) of the MDIs, variations in the thickness of the keratinized mucosa around implants, as well as differences in the thickness of the mucosa at the overdenture’s bearing area, which could account to different levels of stress and strain around implants. The strength of this study is the long period of clinical follow-up (5 years) and the controlled cohort prospective study design.

Overall, the findings of the present study point out that a treatment with only three MDIs is a clinically acceptable treatment option: the same as treatment with four MDIs [2,3,4,5,6,10,11,12,13,14,15,16,33,34,35,36,37,38,39,40,41,45]. 

## 5. Conclusions

Within the limitation of this 5-year prospective cohort study, the results revealed no significant differences between the three- and four-MDI groups in terms of the peri-implant marginal bone loss and success and survival rates. Therefore, treatment with three MDIs retaining the mandibular OD (as a less expensive option) is equally as successful as treatment with four MDIs.

## Figures and Tables

**Table 1:** Mean marginal bone level change (in mm) in the 3- and 4-MDI groups with the significance of the differences between the groups.

MBL Change	Group	N	Mean (in mm)	SD	Minimum (in mm)	Maximum (in mm)	*t*	df	*p*
1 year	3 MDIs	36	−0.21	0.24	0.00	−1.08	0.56	72	0.577 NS
	4 MDIs	38	−0.18	0.30	0.00	−1.53			
3 years	3 MDIs	35	−0.33	0.52	0.00	−3.02	0.61	71	0.544 NS
	4 MDIs	38	−0.26	0.45	0.00	−2.46			
5 years	3 MDIs	34	−0.33	0.27	0.00	−1.00	0.60	69	0.546 NS
	4 MDIs	37	−0.36	0.74	0.00	−4.38			

SD—standard deviation; df—degree of freedom; NS—not significant; *p* < 0.05- is considered significant.

**Table 2:** Survival analysis comparing the 3-MDI and 4-MDI groups throughout five years of supporting mandibular overdentures (implant level).

			Successful Implants		95% Confidence Interval (Time in Months)	
Group	Number of implants	Number of events (failure and compromised survival)	n	Rate (%) (success and satisfactory survival)	Lower bound	Upper bound
3-MDI	108	9 (8.3%)	99	91.7	56.23	50.10
4-MDI	160	10 (6.2%)	150	93.8	57.6	60.17
Overall	268	19 (7.1%)	249	92.9	57.53	59.67

n—number.

**Table 3:** Comparison of the 3-MDI and 4-MDI groups (implant level).

	Chi-Square	df	*p*
Log-rank (Mantel–Cox)	0.373	1	0.541 NS

Test of comparison of survival distributions for the different groups (3- vs. 4-MDI); df—degree of freedom; *p—*significance; NS—not significant (*p* > 0.05).

**Table 4:** Survival analysis comparing the 3-MDI and 4-MDI groups throughout five years of supporting mandibular overdentures at the patient level.

			Successful Implants		95% Confidence Interval (Time in Months)	
Group	Number of implants	Number of events (failure and compromised survival)	n	Rate (%) (success and satisfactory survival)	Lower bound	Upper bound
3-MDI	37	3 (8.1%)	34	91.9	57.58	60.91
4-MDI	40	4 (10.0%)	36	90.0	52.62	61.58
Overall	77	7 (9.1%)	70	90.9	55.85	60.43

n—number.

**Table 5:** Comparison of the 3-MDI and 4-MDI groups (patient level).

	Chi-Square	df	*p*
Log-rank (Mantel–Cox)	0.091	1	0.762 NS

Test of comparison of survival distributions for the different groups (3- vs. 4-MDI); df—degree of freedom; *p—*significance; NS—not significant (*p* > 0.05).

**Table 6:** Overdenture complications and maintenance registered through the 5-year observation period.

OD Complications and Maintenance		
OD relining		
	3-MDI group	4-MDI group
1st year	1	1
3rd year	1	1
5th year	4	3
Metal housing loosening from acrylic resin		
	3-MDI group	4-MDI group
1st year	1	2
3rd year	2	1
5th year	2	3
Retentive elements change		
	3-MDI group	4-MDI group
1st year	7	3
3rd year	34	24
5th year	30	24
Artificial tooth detachment or fracture		
	3-MDI group	4-MDI group
1st year	1	1
3rd year	0	1
5th year	1	1

## Data Availability

The data presented in this study are available on request from the corresponding author. The data are not publicly available due to privacy.
